# Static Plantar-Load Distribution and Stabilometry in Children and Adolescents with Idiopathic Scoliosis Under Different Testing Conditions: A Pilot Study

**DOI:** 10.3390/jcm15176867

**Published:** 2026-09-04

**Authors:** Oana-Cristina Rădulescu, Alina-Daniela Totorean, Andreea-Adriana Neamțu, Laura Maghiar, Oana Suciu, Andreea Niță, Liliana Cațan, Elena Amăricăi

**Affiliations:** 1 Doctoral School, “Victor Babeș” University of Medicine and Pharmacy, 300041 Timișoara, Romania; oana.radulescu@umft.ro; 2 Department of Rehabilitation, Physical Medicine and Rheumatology, Faculty of Medicine, “Victor Babeș” University of Medicine and Pharmacy, 300041 Timișoara, Romania; oanasuciu78@umft.ro (O.S.); nita.andreea@umft.ro (A.N.); catan.liliana@umft.ro (L.C.); amaricai.elena@umft.ro (E.A.); 3 Department of Toxicology, Faculty of Pharmacy, “Victor Babeș” University of Medicine and Pharmacy, Eftimie Murgu Square, No. 2, 300041 Timișoara, Romania; andreea.neamtu@umft.ro; 4 Research Centre for Pharmaco-Toxicological Evaluation, Faculty of Pharmacy, “Victor Babeș” University of Medicine and Pharmacy, Eftimie Murgu Square, No. 2, 300041 Timișoara, Romania; 5 Department of Pathology, Clinical County Emergency Hospital of Arad, Andrenyi Karoly Str., No. 2–4, 310037 Arad, Romania; 6 Department of Pathology, “Pius Brînzeu” Clinical County Emergency Hospital, Liviu Rebreanu Boulevard, No. 156, 300723 Timișoara, Romania; 7 Department of Psycho-Neurosciences and Rehabilitation, Faculty of Medicine and Pharmacy, University of Oradea, Universității Str., No. 1, 410087 Oradea, Romania; 8 Department of Dermatovenerology, Clinical County Emergency Hospital Bihor, Gheorghe Doja Str., No. 65, 410169 Oradea, Romania; 9 Transdisciplinary Research Center in Medical Rehabilitation, Balneology and Rheumatology, “Victor Babeș” University of Medicine and Pharmacy, 300041 Timișoara, Romania; 10 Research Center for Assessment of Human Motion, Functionality and Disability, “Victor Babeș” University of Medicine and Pharmacy, 300041 Timișoara, Romania

**Keywords:** adolescent idiopathic scoliosis, plantar-load distribution, stabilometry, postural control, center of pressure, sensory conditions

## Abstract

**Background/Objectives:** Adolescent idiopathic scoliosis (AIS) is associated not only with structural spinal deformities but also with alterations in postural control and sensorimotor integration. Objective assessment of balance and plantar-load distribution may provide additional insight into functional impairments, particularly in patients with mild and moderate curves. The aim of this study was to compare static plantar-load distribution and stabilometry parameters in children and adolescents with idiopathic scoliosis under different sensory and postural conditions. **Methods:** This pilot study included 19 children and adolescents (18 female, 1 male) diagnosed with mild and moderate AIS. Load distribution and stabilometric parameters were assessed using a capacitive platform under three testing conditions: eyes open, eyes closed, and head extension. Parameters evaluated included load distribution across plantar regions and center-of-pressure-related stability measures. Comparisons were performed between conditions to evaluate the influence of visual and vestibular modulation on postural control. **Results:** Total plantar load did not differ between the right and left foot, and global relative plantar-load distribution remained stable across the three testing conditions. A consistent side-to-side asymmetry was present at the fifth metatarsal head, where load on the left side was significantly lower than on the right in every condition and remained significant after Holm correction for multiple comparisons (*p* = 0.001–0.002; |r| = 0.94–0.99). Higher left-sided load was also observed at the first metatarsal head and heel; the heel asymmetry remained significant after correction only in the eyes-open condition (Holm *p* = 0.044), whereas the remaining differences did not survive correction and are reported as exploratory. The left side corresponded to the side of lumbar convexity. Postural stability differed markedly between conditions: center-of-pressure path length, 90% confidence-ellipse area and maximum center-of-pressure speed were greater under the eyes-closed and head-extension conditions than with eyes open, indicating reduced postural stability when visual input was removed or head position was altered. The two challenge conditions did not differ significantly from each other. **Conclusions:** In this uncontrolled pilot study, children and adolescents with mild-to-moderate idiopathic scoliosis showed a consistent reduction in load under the left fifth metatarsal head, whereas, apart from a higher left heel load that was significant only with eyes open, other regional asymmetries were exploratory. Stabilometric parameters deteriorated under both visual deprivation and head extension, with no statistically significant difference between the two challenge conditions. Because postural changes became apparent mainly when visual or head-position conditions were altered, assessment under such conditions may reveal condition-related changes in postural control within this AIS cohort. However, because no healthy control group was included, these findings should not be interpreted as evidence of AIS-specific sensory-integration deficits. These preliminary findings require confirmation in larger controlled studies including healthy age-matched participants.

## 1. Introduction

Adolescent idiopathic scoliosis (AIS) is a three-dimensional spinal deformity characterized by lateral curvature, vertebral rotation and disturbances in global postural alignment. It typically develops during periods of rapid skeletal growth and remains a multifactorial condition with unclear etiology. In addition to structural spinal changes, increasing evidence suggests that AIS is associated with alterations in neuromuscular control, postural stability, and sensorimotor integration [1,2].

Among different curve patterns, double major or S-shaped scoliosis represents a more complex presentation, involving compensatory thoracic and lumbar curves. This configuration leads to altered biomechanical loading of the spine and trunk, resulting in asymmetric force distribution and compensatory adaptations in both sagittal and coronal planes [2,3]. These characteristics make S-shaped scoliosis more challenging to evaluate and manage clinically compared to single-curve deformities. Recent studies have demonstrated that adolescents with idiopathic scoliosis exhibit significant impairments in postural control compared to healthy individuals. These include reduced balance stability, increased sway during standing tasks, and altered neuromuscular activation patterns. Such findings support the hypothesis that AIS is not only a structural deformity but also a disorder involving dysfunction of global postural regulation systems [4,5].

In this context, postural data analysis has become increasingly relevant in the assessment of scoliosis. Modern evaluation methods extend beyond traditional radiographic measurements and include stabilometric platforms, load distribution analysis, inertial measurement units, and surface asymmetry mapping. These tools allow objective quantification of balance control, center-of-pressure displacement, and postural asymmetries in three-dimensional space [6,7]. Advances in motion analysis technologies and computational modeling have significantly improved the ability to assess spinal and postural behavior in AIS. Techniques such as inertial sensor-based gait analysis and surface reconstruction algorithms now provide non-invasive and reproducible measures of spinal alignment and functional mobility [3,7,8]. These developments enhance the clinical value of postural data analysis in both diagnosis and follow-up. Given that postural stability relies on the integration of visual, vestibular, and proprioceptive inputs, alterations in sensory conditions may unmask subtle deficits in postural control in individuals with AIS. Therefore, assessing stabilometric parameters under different sensory conditions may provide additional insight into the mechanisms of postural adaptation.

Despite this growing interest, most studies of postural control in adolescent idiopathic scoliosis have assessed standing balance only with the eyes open, and few have systematically examined how the controlled withdrawal or alteration of individual sensory channels affects postural stability in this population. Manipulating sensory input is informative because it helps isolate the relative contribution of each system to balance: closing the eyes removes visual feedback and increases reliance on the somatosensory and vestibular systems, whereas head extension alters cervical proprioceptive and vestibular input while vision remains available. Comparing these conditions may therefore reveal compensatory strategies that remain hidden during conventional eyes-open assessment.

Recent evidence suggests that adolescents with idiopathic scoliosis exhibit deficits in sensory integration and postural control, particularly under sensory-challenging conditions such as visual deprivation or vestibular perturbation [5,9,10]. However, existing studies have relied predominantly on force-plate posturography and have rarely combined stabilometric assessment with static plantar-pressure mapping [9,11]. Furthermore, the effects of head extension, a maneuver known to alter cervical proprioceptive and vestibular input, have not been specifically investigated under head-extension conditions in AIS populations [12,13,14]. Therefore, the combined assessment of plantar-load distribution and stabilometric parameters under visual and cervical-proprioceptive challenges represents an important gap in the current literature [9,11].

The objectives of our study were to assess and compare the static plantar-load distribution and stabilometry parameters in children and adolescents with mild-to-moderate idiopathic scoliosis when tested in different conditions. We hypothesized that altered sensory conditions (visual deprivation and head extension) would reveal greater changes in stabilometric parameters than the conventional eyes-open condition and that regional plantar-load asymmetries would remain detectable across testing conditions. We chose three distinct situations, namely with eyes open, eyes closed and head extension. Our hypothesis was that there will be differences in stabilometry with a greater postural stability when tested with eyes open.

The principal focus of this pilot study was the comparison of stabilometric parameters (center-of-pressure path length, 90% confidence-ellipse area, and maximum center-of-pressure speed) across the three testing conditions (eyes open, eyes closed, and head extension). We hypothesized that postural stability would be greatest during the eyes-open condition and would deteriorate under sensory-challenging conditions. Load distribution was considered an exploratory outcome because of the limited evidence regarding regional plantar-loading adaptations in adolescents with idiopathic scoliosis, particularly under different sensory conditions.

## 2. Materials and Methods

This study included patients diagnosed with mild-to-moderate idiopathic dextroconvex thoracic and sinistroconvex lumbar scoliosis. Written informed consent was obtained from all participants who were of legal age and from the parents or legal guardians of minors; in addition, age-appropriate assent was obtained from the minors. All participants received an information leaflet describing the study and were informed of their right to withdraw at any time without consequences. The study was conducted in accordance with the principles of the Declaration of Helsinki and was approved by the Institutional Ethics Committee of the Louis Țurcanu Children’s Emergency Hospital (No. 173/2024, approved on 13 January 2025) and the Ethics Committee of the Victor Babeș University of Medicine and Pharmacy, Timișoara (No. 52/4 November 2024). Participants were recruited from January 2025 to May 2026 from the Department of Pediatric Surgery and the Pediatric Rehabilitation Unit of the Louis Țurcanu Children’s Emergency Hospital, Timișoara, Romania.

### 2.1. Participants

A total of 19 adolescents were enrolled in the study, including 18 females and 1 male, resulting in a substantially uneven sex distribution (Table 1). All eligible participants who agreed to participate completed the study. No participant withdrew after enrolment, and no datasets were excluded from the final analysis. Because this was designed as a pilot study, no formal a priori sample size calculation was performed. The sample size was determined by feasibility and corresponded to all eligible participants who met the inclusion criteria and agreed to participate during the recruitment period. The primary objective was to obtain preliminary data to evaluate the feasibility of the study protocol and to estimate effect sizes for future adequately powered studies. Participants were between 12 and 19 years of age (mean age: 15.3 ± 1.8 years). The average height was 162.5 ± 6.8 cm (range: 150–173 cm), the average body weight was 48.5 ± 6.9 kg (range: 38–62 kg), and the mean body mass index (BMI) was 17.6 ± 1.8 kg/m^2^ (range: 15.37–21.87 kg/m^2^). All participants were otherwise healthy adolescents diagnosed with idiopathic scoliosis and without additional musculoskeletal or neurological disorders. The pronounced predominance of female participants (18 females and one male) should be considered an important study limitation. This distribution precluded any meaningful assessment of sex-related differences and limits the generalizability of the findings, particularly to male adolescents with idiopathic scoliosis.

#### 2.1.1. Inclusion Criteria

The study included children and adolescents aged 10–19 years diagnosed with mild-to-moderate idiopathic scoliosis, characterized by dextroconvex thoracic and sinistroconvex lumbar curvature patterns. Diagnosis was confirmed through both clinical examination and radiological assessment. Curve severity was quantified on standing posteroanterior spinal radiographs using the Cobb method, with mild scoliosis defined as a Cobb angle of 10–25° and moderate scoliosis as 26–40°. Radiographic evaluation and Cobb angle measurement were performed at the referring clinic as part of routine care and served to confirm eligibility; the individual Cobb angle values remained in the paper-based clinical records of the referring clinic and were not transferred to the pseudonymized study dataset. Participation in the study was voluntary, and informed consent was obtained from all participants and/or their legal guardians prior to enrollment.

#### 2.1.2. Exclusion Criteria

Participants were excluded if they had undergone lower-limb orthopedic surgery within the previous three months, exhibited disorders affecting static balance or gait, or had neurological conditions influencing balance, posture, or walking ability. Individuals with cognitive impairments or limited ability to understand and cooperate with study procedures were also excluded. Before enrollment, all participants underwent routine clinical evaluation and medical history review. Only otherwise healthy adolescents with idiopathic scoliosis were included; therefore, none of the enrolled participants had known neurological, vestibular, visual, or additional musculoskeletal disorders that could reasonably affect postural stability.

### 2.2. Measurements

Static plantar-load distribution and stabilometric assessment were performed using the PoDATA 2.0 platform (Chinesport, Udine, Italy). The system quantifies the distribution of body weight across predefined plantar regions of each foot, including the first metatarsal head, fifth metatarsal head, and calcaneus, and simultaneously calculates stabilometric variables derived from center-of-pressure displacement. The manufacturer reports regional loading as body weight distributed under each plantar region, whereas the present study analyzed these data as the percentage distribution of plantar load across the predefined anatomical regions. Accordingly, throughout this manuscript the term “plantar-load distribution” is used instead of “plantar pressure”, because the device does not report pressure in physical units (e.g., kPa or N/cm^2^) [15]. Each participant underwent an evaluation comprising three 20 s static recordings (approximately 5 min in total, including instructions, familiarization, and rest intervals), one for each condition: eyes open, eyes closed, head extension. The testing conditions were performed in the same predefined sequence for all participants: eyes open, followed by eyes closed, and finally head extension. The order of testing was not randomized. The method was non-invasive, practical, and easy to apply. The PoDATA 2.0 device enables the evaluation of plantar-load distribution and force distribution during static stance, providing quantitative data on plantar support and allowing the identification of plantar overloads, postural rotations, and asymmetries [15]. Children were instructed to stand barefoot on the platform in an upright position, with the lower limbs fully extended and the arms relaxed alongside the body. During each 20 s recording, participants stood still. In the eyes-open condition, participants fixated a target on the wall directly ahead (a circular visual target positioned at eye level, approximately 2 m in front of the platform). Participants who habitually wore visual correction (glasses or contact lenses) kept it on during all testing conditions. In the eyes-closed condition, participants kept their eyes closed. During the head-extension condition, participants were instructed to keep their eyes open and to extend only the head and neck backwards as far as comfortably possible while maintaining the trunk in an upright position. No visual target was provided during this condition, and the head-extension angle was not objectively measured or standardized. Foot placement was standardized, with the feet positioned at an angle of approximately 30° between their longitudinal axes and the heels separated by 5 cm, in accordance with a commonly used standardized stabilometric stance [15,16]. This standardized position was selected to ensure consistent foot placement across participants and to minimize variability in plantar-load distribution and stabilometric measurements. Participants were instructed to remain still, avoid speaking, and, during the eyes-open condition, to maintain their gaze on the target throughout the trial. A trial was deemed invalid and repeated if any of the following occurred: movement or elevation of one or both arms, lifting of the forefoot or heel, loss of the prescribed standing position, head movement, or verbalization. Before data acquisition, all participants received standardized verbal instructions regarding the testing procedure and the required body position to ensure that they understood the assessment protocol. In addition, a brief familiarization period in the standardized testing position on the platform was allowed before the first recording. Each testing condition was represented by one valid 20 s recording. Invalid recordings were repeated immediately until a valid trial was obtained. Invalid recordings were infrequent and typically required no more than one repetition per condition. Short rest intervals of approximately 30–60 s were provided between testing conditions to allow participant repositioning and delivery of the instructions for the subsequent task. No adverse events, falls, or losses of balance occurred during any of the testing sessions. The system was calibrated according to the manufacturer’s instructions before each testing session. All values in the final dataset were verified against the original device reports. One transcription error (a left-foot load recorded as 196% under the eyes-closed condition) was detected through an internal consistency check, as bilateral foot loads should sum to approximately 100%, and was corrected to the value in the original report (49%); no other values required correction. Because foot and regional loads are exported as separately rounded integer percentages, bilateral sums may deviate from 100% by a few percentage points, which reflects rounding rather than data errors. The apparently identical stabilometric values of two participants under the eyes-closed condition were also re-checked against the original device reports and confirmed to be correct. Following this data verification, all summary statistics, statistical tests, effect sizes, and figures were calculated from the verified dataset, and all numerical values reported in the manuscript are based on this dataset; the complete individual-level data are provided in Supplementary Table S1 to ensure full transparency. Participant recruitment and testing were completed in two consecutive acquisition batches because enrolment extended over the study period. Both batches were assessed using the same PoDATA 2.0 platform, identical software version, calibration procedure, assessor, standardized testing protocol, examination room, and environmental conditions. No changes were made to the acquisition protocol or data-processing procedures between the two batches. Data acquisition and calculation of plantar-load distribution and center-of-pressure variables were performed using the proprietary PoDATA 2.0 software supplied by the manufacturer. Detailed technical specifications, including the sensor configuration, sampling frequency, sensor resolution, signal-processing algorithms, and the internal calculation procedures for center-of-pressure variables, are proprietary and are not disclosed by the manufacturer. For the present analyses, center-of-pressure (CoP) path length was defined as the total distance traveled by the CoP during the 20 s recording, the 90% confidence-ellipse area as the area of the ellipse containing 90% of the recorded CoP positions, and maximum CoP speed as the highest instantaneous CoP displacement velocity during the trial, as reported by the device software. The same capacitive PoDATA platform, with an identical 20 s acquisition protocol and the same outcome definitions, has previously been used for static plantar-load and stabilometric assessment in adolescents with idiopathic scoliosis [17]. The percentage distribution of load across the three plantar regions of each foot was calculated for each participant. According to the reference model of an ideal load distribution, the load of each foot should be distributed as follows: 16.67% on the fifth metatarsal head, 33.33% on the first metatarsal head, and 50.00% on the heel [15].

### 2.3. Statistical Analysis

Normality was assessed with the Shapiro–Wilk test, which indicated departures from normality for several variables; given the small sample size, the skewed distributions and the presence of outliers, non-parametric tests were used. Outliers were identified by inspection of the individual data distributions; because all values were verified against the original device reports, every observation was retained in the analyses. Given the skewed distributions, medians with interquartile ranges are reported alongside means and standard deviations in Table 2 and Table 3. Center-of-pressure path length was considered the stabilometric variable of principal interest, as it is one of the most widely used indicators of postural stability. Because it was not formally prespecified before data analysis, it should be regarded as a candidate primary outcome for future confirmatory studies rather than a prespecified primary outcome. The primary comparison was the within-subject comparison of stabilometric variables across the three testing conditions (eyes open, eyes closed, and head extension). Holm correction was applied separately to each predefined family of related post hoc comparisons: (1) regional right-versus-left plantar-load comparisons within each testing condition and (2) pairwise post hoc comparisons following significant Friedman tests for each variable. Analyses of regional plantar-load changes across testing conditions were considered secondary. Regional findings that did not remain significant after Holm correction were regarded as exploratory and hypothesis-generating. For same-site comparisons between testing conditions, post hoc pairwise comparisons were Holm-adjusted when the corresponding Friedman omnibus test was significant; for sites with a non-significant omnibus test, pairwise *p*-values are reported as unadjusted, exploratory values. Right-versus-left (paired) comparisons at each plantar region were performed with the Wilcoxon signed-rank test and corrected for multiple comparisons across the family of regional side-to-side tests using the Holm procedure. Differences across the three related conditions (eyes open, eyes closed and head extension) were assessed primarily with the Friedman test as the omnibus repeated-measures test; when the omnibus test was significant, post hoc pairwise comparisons were performed with the Wilcoxon signed-rank test and Holm-adjusted. Effect sizes for paired comparisons were expressed as the matched-pairs rank-biserial correlation (*r*) with 95% bootstrap confidence intervals (5000 iterations), and Kendall’s coefficient of concordance (*W*) was reported as the effect size for the Friedman test. In addition, a post hoc sensitivity analysis, which was not prespecified, repeated the principal analyses after excluding the three participants assessed in the second acquisition batch (*n* = 16), in order to examine the robustness of the findings to the two-batch recruitment structure. All statistical analyses were performed using Microsoft Excel (version 16.83, Microsoft 365 for macOS), GraphPad Prism (version 10.1.2 for macOS), and R Studio (version 2025.05.0+496). The significance threshold was set at *p* < 0.05 throughout, unless otherwise specified.

## 3. Results

Plantar-load distribution for the three conditions is shown in Table 2. Total load on the right and left foot did not differ in any condition. Among the regional side-to-side comparisons, the load on the left fifth metatarsal head was consistently and substantially lower than on the right and remained significant after Holm correction in every condition (Holm *p* = 0.001–0.002; |r| = 0.94–0.99, 95% CIs 0.77–1.00). The higher left-sided load at the first metatarsal head (raw *p* = 0.014–0.045) did not survive Holm correction (Holm *p* = 0.056–0.085) and is therefore reported as exploratory. The higher left-sided load at the heel (raw *p* = 0.007–0.043) remained significant after Holm correction only in the eyes-open condition (Holm *p* = 0.044; |r| = 0.70, 95% CI 0.27–1.00); under the eyes-closed and head-extension conditions it did not survive correction (Holm *p* = 0.056–0.085) and is reported as exploratory. In all participants the left side corresponded to the side of lumbar convexity.

When comparing the plantar-load distribution of the same site across measurement conditions (Holm-adjusted post hoc comparisons for the left first metatarsal head and left heel, whose Friedman omnibus tests were significant; unadjusted exploratory *p*-values otherwise), there were no significant differences between the eyes-open and eyes-closed testing for the right foot load (*p* = 0.47), left foot load (*p* = 0.30), right first metatarsal head (*p* = 0.13), right and left fifth metatarsal head (*p* = 0.16 and *p* = 0.13, respectively), and right heel (*p* = 0.08). The load on the left heel was decreased (Holm-adjusted *p* = 0.029; |*r*| = 0.74) and the load on the left first metatarsal head was increased (Holm-adjusted *p* = 0.034; |*r*| = 0.73) with eyes closed.

When analyzing the plantar-load distribution of the same location in the eyes-open and head-extension conditions, there were no significant differences for the right foot load (*p* = 0.74), left foot load (*p* = 0.98), right first metatarsal head (*p* = 0.07), right and left fifth metatarsal head (*p* = 0.98 and *p* = 0.29, respectively), and right heel (*p* = 0.30). However, the load on the left first metatarsal head was increased (Holm-adjusted *p* = 0.034; |*r*| = 0.69) and the load on the left heel was decreased (Holm-adjusted *p* = 0.029; |*r*| = 0.67) with head extension compared with eyes-open testing.

Comparing the plantar-load distribution of the same site in the eyes-closed and head-extension conditions, there were no significant differences for the right foot load (*p* = 0.36), left foot load (*p* = 0.50), right first metatarsal head (*p* = 0.18), right and left fifth metatarsal head (*p* = 0.55 and *p* = 0.35, respectively), and right and left heel (*p* = 0.76 and *p* = 0.70, respectively). In contrast, the load on the left first metatarsal head was increased (Holm-adjusted *p* = 0.043; |*r*| = 0.59) with head extension compared with eyes-closed testing.

Stabilometric data for the three conditions are presented in Table 3. The Friedman test was significant for all three parameters (center-of-pressure path length, 90% confidence-ellipse area and maximum center-of-pressure speed; all omnibus *p* ≤ 0.002). Holm-adjusted post hoc comparisons showed that each parameter was significantly higher under both the eyes-closed and head-extension conditions than with eyes open (Holm *p* = 0.001–0.032), with large effect sizes (path length: |r| = 0.76 [95% CI 0.39–1.00] vs. eyes closed and 0.63 [0.19–0.99] vs. head extension; ellipse area: 0.67 [0.24–1.00] and 0.71 [0.26–0.98]; maximum speed: 0.86 [0.56–1.00] and 0.73 [0.34–0.99]). These results indicate better postural stability with eyes open than under either challenge condition.

The eyes-closed and head-extension conditions did not differ from each other for any stabilometric parameter (Holm *p* ≥ 0.12); although the mean values were numerically higher under eyes-closed than under head-extension testing, this difference was not significant.

**Figure 1:**
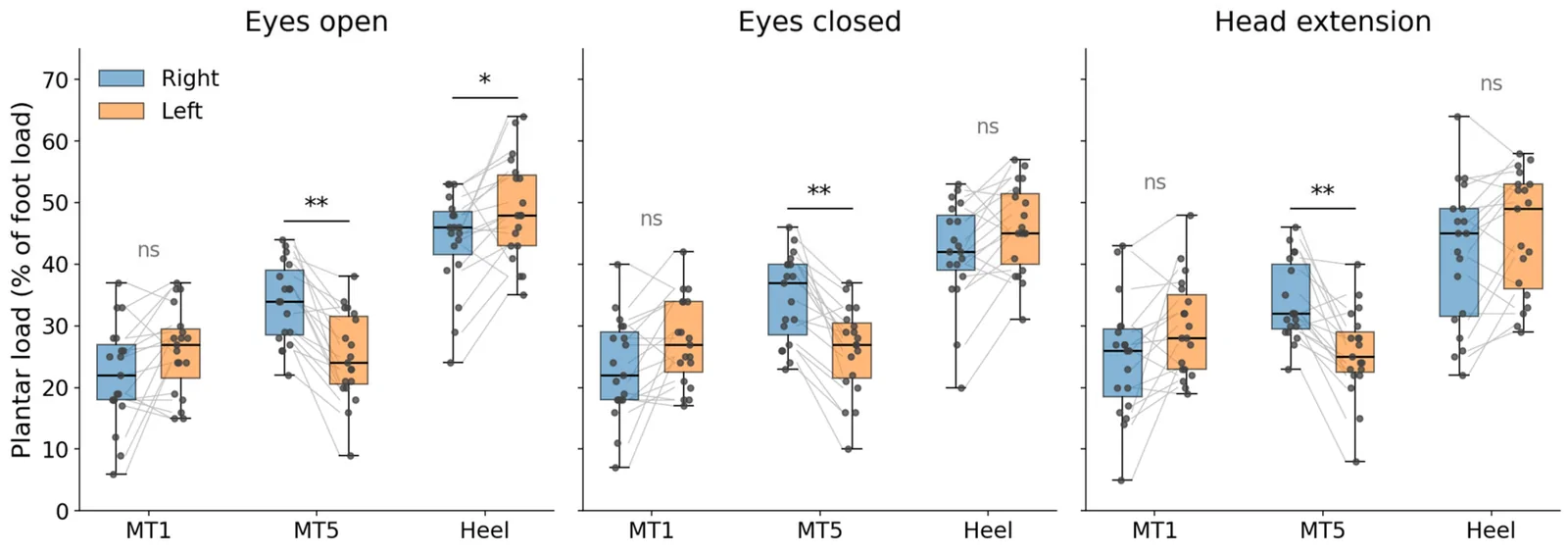
Right-versus-left plantar load at the first metatarsal head (MT1), fifth metatarsal head (MT5) and heel under the three testing conditions (eyes open, eyes closed, head extension). Boxes show the median and interquartile range, whiskers indicate the full range, individual points show single participants, and gray lines connect the paired right–left observations of each participant. Asterisks denote side-to-side differences that remained significant after Holm correction across the regional family (* Holm *p* < 0.05, ** Holm *p* < 0.01); ns, not significant after correction.

**Figure 2:**
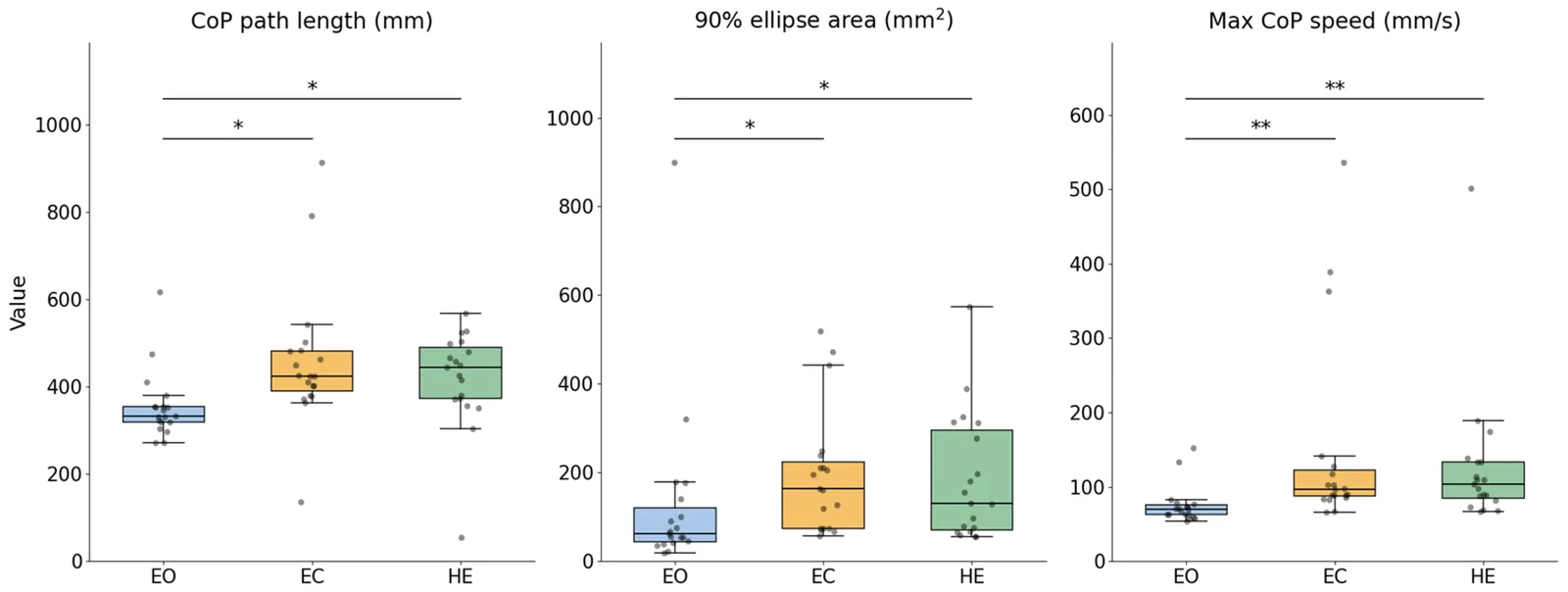
Stabilometric parameters (center-of-pressure [CoP] path length, 90% confidence-ellipse area and maximum CoP speed) across the eyes-open (EO), eyes-closed (EC) and head-extension (HE) conditions. Boxes show the median and interquartile range with individual values; brackets indicate Holm-corrected post hoc Wilcoxon comparisons (* *p* < 0.05, ** *p* < 0.01).

**Figure 3:**
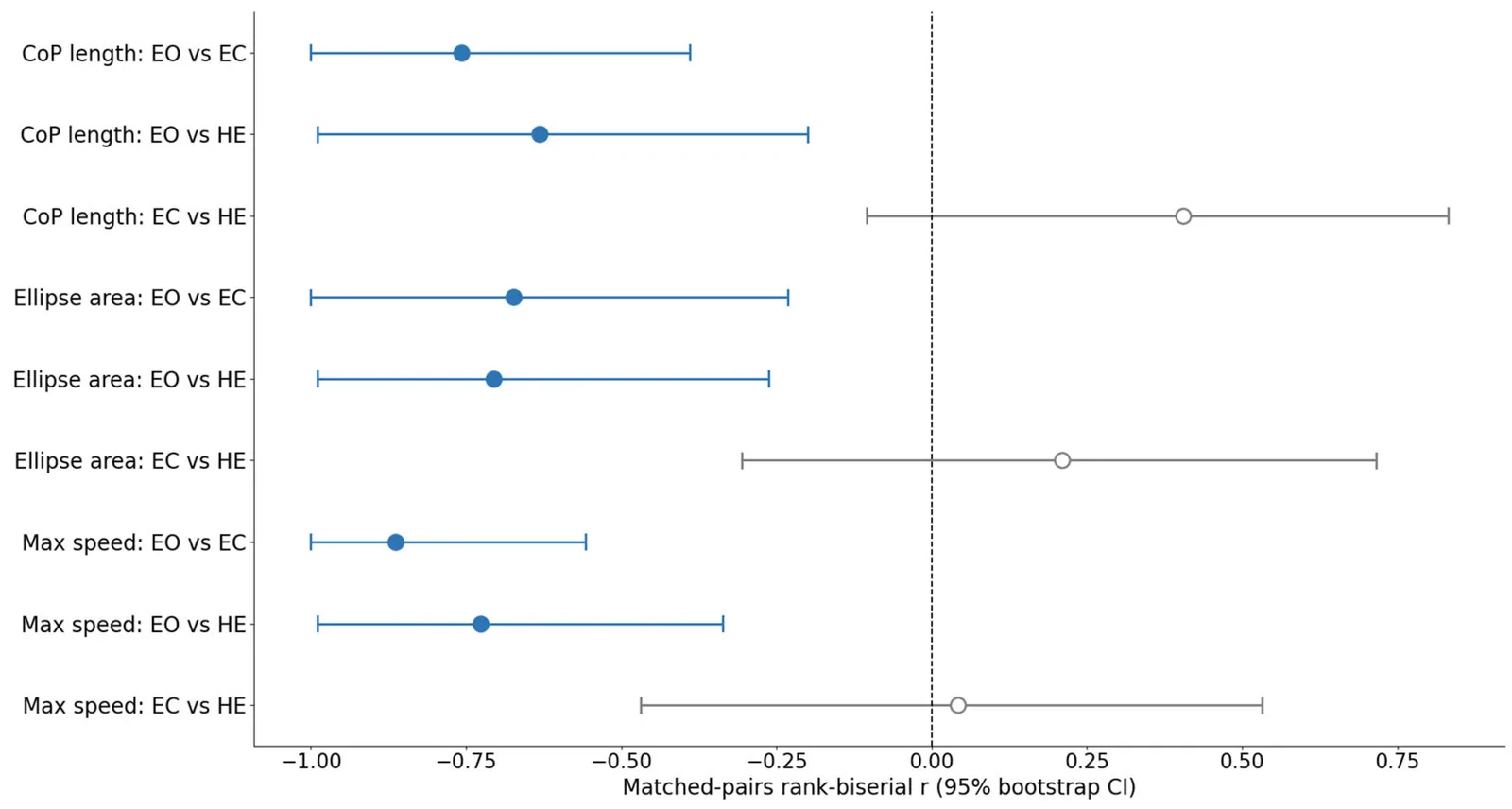
Effect sizes (matched-pairs rank-biserial correlation r with 95% bootstrap confidence intervals) for the post hoc comparisons between conditions. Filled markers indicate comparisons remaining significant after Holm correction (*p* < 0.05); the dashed line marks a null effect (*r* = 0). The rank-biserial correlation ranges from −1 to +1; values farther from 0 indicate larger and more consistent differences between the two compared conditions, and markers whose confidence interval does not cross the dashed line correspond to differences that are unlikely to be due to chance.

Repeated-measures effects across the three conditions were tested with the Friedman omnibus test (Figure 1, Figure 2 and Figure 3). Global right/left foot loading did not change across conditions, whereas significant condition effects emerged for the left first metatarsal head (χ^2^(2) = 9.72, *p* = 0.008, Kendall *W* = 0.26), the left heel (χ^2^(2) = 11.08, *p* = 0.004, *W* = 0.29) and all three stabilometric parameters (center-of-pressure path length: χ^2^(2) = 15.47, *p* < 0.001, *W* = 0.41; 90% confidence-ellipse area: χ^2^(2) = 13.05, *p* = 0.002, *W* = 0.34; maximum center-of-pressure speed: χ^2^(2) = 15.89, *p* < 0.001, *W* = 0.42). The corresponding Holm-adjusted post hoc comparisons for the left first metatarsal head and the left heel are reported in the same-site analyses above. Holm-adjusted post hoc comparisons confirmed that center-of-pressure path length, ellipse area and maximum speed were significantly greater under both the eyes-closed and head-extension conditions than with eyes open (all Holm *p* < 0.05; rank-biserial r 0.63–0.86), whereas the two challenge conditions did not differ (Holm *p* ≥ 0.12). For the regional side-to-side comparisons, the reduction in left fifth-metatarsal load survived Holm correction across the regional family in every condition (Holm *p* = 0.001–0.002; |r| = 0.94–0.99), and the higher left heel load survived correction only in the eyes-open condition (Holm *p* = 0.044); the first-metatarsal and the remaining heel asymmetries did not (Holm *p* = 0.056–0.085) and are reported as exploratory. In a sensitivity analysis excluding the three participants assessed in the second acquisition batch (n = 16), the pattern of principal findings was unchanged: the fifth-metatarsal asymmetry remained significant in every condition (Holm *p* = 0.005), the eyes-open heel asymmetry remained significant (Holm *p* = 0.015), all stabilometric condition effects persisted (Friedman *p* ≤ 0.0002; eyes-open versus challenge comparisons Holm *p* ≤ 0.014), and no significant difference emerged between the eyes-closed and head-extension conditions.

## 4. Discussion

In this uncontrolled pilot study of children and adolescents with mild-to-moderate S-shaped idiopathic scoliosis, total plantar loading and overall right/left foot balance remained stable across the three conditions, while two findings stood out. First, a consistently lower load under the left fifth metatarsal head was observed across all testing conditions within this sample and survived correction for multiple comparisons in every condition; a higher left heel load additionally survived correction in the eyes-open condition only. Second, center-of-pressure path length, ellipse area and speed increased when vision was removed or the head was extended, with no statistically significant difference in destabilization between the two challenge conditions. Because this was a pilot investigation, these observations are preliminary and motivate evaluation in a larger sample. Double-curve scoliosis is commonly associated with compensatory mechanisms that help maintain global spinal alignment and preserve the body’s center of gravity, potentially contributing to a relatively stable overall plantar-load distribution despite regional asymmetries [18,19].

In cases of double-curve scoliosis, plantar-load distribution provides a more comprehensive representation of overall postural reorganization and the associated complex compensatory mechanisms. Conversely, in single-curve scoliosis, plantar-load distribution characteristics may primarily reflect the effects of unilateral trunk displacement [20]. Spinal deformities can also alter lower-limb kinematics during gait, indicating that the postural adaptations associated with scoliosis extend beyond the trunk [21]. A consistent finding across all testing conditions was the significantly lower plantar-load distribution beneath the left fifth metatarsal head compared with the contralateral side. This asymmetry may reflect unequal weight-distribution strategies, which are commonly observed in individuals presenting with spinal alignment abnormalities; however, because assessments were cross-sectional and performed at a single time point, it cannot be determined whether this pattern represents a persistent postural adaptation. Our findings are consistent with those reported by Cațan et al. [17], who observed plantar-pressure asymmetries in female adolescents with moderate idiopathic scoliosis. Similar to their results, we identified persistent asymmetrical plantar loading, suggesting the presence of compensatory postural adaptations within this cohort. Together, these findings suggest that plantar-load assessment may provide complementary quantitative information in adolescents with idiopathic scoliosis; however, in the absence of reliability, minimal-detectable-change, and clinical outcome data, its value as an objective tool for functional evaluation remains to be established.

Because the left side corresponded to the lumbar convexity in our cohort, the observed reduction in load under the left fifth metatarsal head may be compatible with a lateral trunk shift toward the concavity. However, trunk displacement was not directly measured, and because every participant had the same curve direction, anatomical side and curve direction are completely confounded. Therefore, the present design cannot establish a causal relationship between lumbar convexity and the observed plantar-loading pattern. The persistence of the fifth-metatarsal asymmetry across all three sensory conditions may reflect a relatively stable plantar-loading pattern rather than a transient sensory-dependent response. However, because trunk alignment, compensatory movements, and sensory integration were not directly assessed, all mechanistic interpretations should be regarded as hypotheses requiring confirmation in future studies.

Head extension was associated with increased center-of-pressure path length and speed compared with the eyes-open condition. Rather than isolating a single sensory channel, extending the head and neck simultaneously alters visual orientation, vestibular stimulation and cervical proprioception, so the resulting destabilization is best interpreted as a combined sensorimotor challenge rather than a specific cervical-proprioceptive effect [12,22]. Altered head-and-neck posture is itself clinically relevant, since sustained postural deviations such as forward head posture can alter cervical muscle function and may benefit from targeted multidisciplinary rehabilitation [23]. Notably, no statistically significant differences were detected between the eyes-closed and head-extension conditions. Because the present pilot study was not designed or powered to demonstrate equivalence, these findings should not be interpreted as indicating equivalent effects of the two sensory challenges.

Visual deprivation significantly increased center-of-pressure (CoP) path length and maximum CoP speed compared with the eyes-open condition. These findings are consistent with the well-established contribution of visual input to postural control reported in previous studies. However, because no healthy control group was included, the present study cannot determine whether the magnitude of these condition-related responses differs from that expected in healthy adolescents or is specific to adolescent idiopathic scoliosis. In the absence of visual input, the central nervous system places greater reliance on somatosensory and vestibular cues, resulting in increased postural sway [24,25]. These mechanisms are well established in healthy populations and are discussed here to provide physiological context rather than as mechanisms demonstrated directly by the present study [24,25].

Localized changes in plantar-load distribution revealed increased loading of the left first metatarsal head in both the eyes-closed and head-extension conditions, accompanied by a reduction in left heel loading during the eyes-closed and head-extension conditions. These condition-related differences remained statistically significant after Holm adjustment of the corresponding post hoc comparisons (adjusted *p* = 0.029–0.043). Nevertheless, they derive from secondary analyses of small regional load shifts in a small, uncontrolled sample and should be regarded as hypothesis-generating. Consequently, any interpretation in terms of compensatory postural strategies should be considered tentative. Because similar adaptations may also occur in healthy individuals exposed to the same sensory challenges, the present study cannot determine whether these responses are specific to adolescent idiopathic scoliosis. Future studies including larger cohorts and healthy control groups are needed to determine whether these regional plantar-load patterns represent reproducible biomechanical adaptations or reflect random variation within small samples. Overall, the present findings indicate that global plantar loading remained relatively stable within this cohort of adolescents with idiopathic scoliosis, whereas postural stability changed under altered sensory conditions. Because healthy adolescents were not evaluated, these observations should be interpreted as condition-related responses within the AIS cohort rather than evidence of AIS-specific alterations in postural responses. Furthermore, because associations with curve severity, patient symptoms, and functional outcomes were beyond the scope of this pilot study, the present findings should not be interpreted as demonstrating clinically meaningful alterations in postural function. From a clinical perspective, and pending confirmation in controlled studies, combined plantar-load and stabilometric assessment under altered visual and head-position conditions may eventually complement routine clinical evaluation in adolescent idiopathic scoliosis, for example as a simple, non-invasive adjunct for monitoring postural control during follow-up or rehabilitation; at present, such applications remain hypothetical and require validation against clinical outcomes. These findings reinforce the concept of multisensory integration and sensory reweighting as fundamental mechanisms underlying the control of upright posture [24,25].

Plantar-load and stabilometric measures may also be relevant to questions about physical activity and sports participation, which are frequently raised by adolescents with idiopathic scoliosis and their families. The present cohort was neither a post-injury nor a post-operative population, and no rehabilitation, functional, or sport-related outcomes were recorded; the present data therefore cannot inform return-to-sport decisions directly. Conceptually, however, the parameters assessed here correspond to the balance and movement-quality domains that criterion-based frameworks recommend be evaluated together with physical capacity and sport-specific readiness, rather than relying on elapsed time alone [26,27]. This consideration is particularly pertinent in idiopathic scoliosis, where a graded resumption of sport is broadly supported by expert consensus but no condition-specific readiness battery has yet been validated [27,28]. In this context, the observation that differences in postural stability emerged only when vision or head position was altered, whereas quiet-standing plantar loading remained comparatively stable, suggests that assessment restricted to a single unchallenged standing condition may underestimate postural-control demands; should this be confirmed in controlled studies, sensory-challenged stabilometry might contribute one objective and low-burden component to such multidimensional assessments. Any such application would first require the test–retest reliability and minimal detectable change of the measurement system to be established and an association with functional and sport-related outcomes to be demonstrated; until then, these considerations remain hypothetical.

### 4.1. Future Directions

Future studies should include larger, adequately powered cohorts together with healthy age-matched control participants to determine whether the observed plantar-load and stabilometric responses are specific to adolescent idiopathic scoliosis. Standardization of the head-extension angle, randomization of the testing order, and acquisition of repeated measurements under each condition would improve methodological rigor and permit assessment of measurement variability. Future investigations should also include participants with different curve patterns and opposite curve directions, together with more comprehensive clinical characterization, including Cobb angle, Lenke classification, skeletal maturity, treatment status, symptoms, and functional outcomes. Finally, validation of the findings using force-platform systems with established measurement properties would strengthen confidence in the clinical interpretation of plantar-load and center-of-pressure measurements. Such studies should also account for the intrinsic variability of plantar-load and center-of-pressure patterns across tasks, contexts, and developmental stages [29,30]. Among the stabilometric variables evaluated, center-of-pressure path length demonstrated the most consistent condition-related differences and may therefore represent a suitable candidate primary outcome for future controlled studies. As an illustration, detecting a between-group difference in center-of-pressure path length of moderate-to-large standardized magnitude (Cohen’s d = 0.6–0.8) with 80% power at a two-sided alpha of 0.05 would require approximately 26–46 participants per group with a non-parametric two-sample comparison; the large within-subject effects observed here (|r| = 0.63–0.86) suggest that condition-related differences of this order are realistic targets for a definitive study.

### 4.2. Limitations

This pilot study has several limitations that should be considered when interpreting the results. First, the small sample size and the pronounced predominance of female participants (18 females and one male) limited the generalizability of the findings. In particular, the inclusion of only one male participant precluded any meaningful evaluation of sex-related differences, and the present results should therefore be interpreted primarily as representative of female adolescents with idiopathic scoliosis. A sensitivity analysis excluding the single male participant could not be performed because sex was not linked to the individual records of the pseudonymized analysis dataset. In addition, the cohort spanned a wide developmental age range (12–19 years); because postural control, vestibular function, and foot morphology evolve across adolescence, pooling data across this range may mask age-related effects, and age-stratified analyses will require larger samples. Second, the uncontrolled pilot design and the absence of a healthy, age-matched control group mean that it cannot be established whether the observed plantar pattern is specific to idiopathic scoliosis or partly reflects side-to-side differences present in the general population, and no causal relationship between scoliosis and the plantar or postural findings can be inferred. In addition, detailed clinical characteristics, including complete Cobb angle distribution, Lenke classification, skeletal maturity, and treatment status (e.g., bracing or scoliosis-specific exercises), were not systematically available for all participants. Consequently, the potential influence of these factors on plantar-load distribution and postural control could not be evaluated. These data are held in the paper-based clinical files of the referring institution and were not available in the pseudonymized study dataset; consequently, neither the Cobb angle distribution nor the relationship between curve magnitude and the plantar-load or stabilometric parameters could be analyzed. This relationship warrants dedicated analysis in future, adequately characterized cohorts, particularly because a previous investigation using the same platform reported significant correlations between the lumbar Cobb angle and fifth-metatarsal load as well as stabilometric parameters [17]. Third, because all participants had the same curve direction, side and curve direction are confounded, so the association between left lumbar convexity and left-sided loading is descriptive only. Fourth, although the principal comparisons were Holm-corrected, the regional side-to-side analyses examined several subregions in only 19 participants; apart from the consistent reduction in left fifth-metatarsal load and the higher left heel load in the eyes-open condition, the regional asymmetries did not survive correction and should be regarded as exploratory. Fifth, assessments were performed at a single time point, preventing analysis of how these biomechanical changes evolve over time. Finally, several protocol parameters were not standardized or formally assessed, including the precise head-extension angle, the order of testing conditions, and test–retest reliability. Because the testing conditions were performed in a fixed, non-randomized sequence, potential order effects, including learning, adaptation, or fatigue, cannot be excluded. In addition, the degree of cervical extension was determined according to each participant’s comfort and was not objectively quantified, which may have contributed to inter-individual variability. Each testing condition was represented by a single valid recording. Although invalid trials were repeated when necessary, repeated valid recordings were not averaged; therefore, some trial-to-trial variability may not have been captured. Several stabilometric variables, particularly the confidence-ellipse area, showed marked between-participant variability; this motivated the non-parametric analysis and the additional reporting of medians with interquartile ranges, and it implies that the mean values should be interpreted with caution. External factors such as individual variation in posture may also have influenced the results. These aspects should be addressed in future, adequately powered studies.

The absence of major plantar-load distribution changes should not necessarily be interpreted as normal postural control, as previous studies have shown that balance impairments in adolescent idiopathic scoliosis become more evident under sensory-challenged conditions than during quiet standing, suggesting the presence of compensatory postural strategies [31,32]. In addition, the present study was not designed to evaluate the test–retest reliability or measurement error of the PoDATA 2.0 system. Consequently, it could not be determined whether the observed differences exceeded the expected measurement error or any minimal detectable change, and therefore their clinical significance could not be established. Furthermore, the proprietary software does not provide publicly available information regarding the internal algorithms used for center-of-pressure calculations. These limitations should be considered when interpreting the present findings and require confirmation in future studies using force-platform systems with published measurement properties.

## 5. Conclusions

In this uncontrolled pilot study of children and adolescents with mild-to-moderate S-shaped idiopathic scoliosis, total plantar load did not differ between the right and left foot. The only regional side-to-side asymmetry that survived correction for multiple comparisons was a consistently lower load under the left fifth metatarsal head, present in all three conditions, together with a higher left heel load that survived correction only in the eyes-open condition; the higher left-sided load at the first metatarsal head did not survive correction and is considered exploratory. Stabilometric parameters (center-of-pressure path length, ellipse area and maximum speed) were significantly higher under both the eyes-closed and head-extension conditions than with eyes open, indicating reduced postural stability when visual input was removed or head position was altered, with no difference between the two challenge conditions. Because condition-related changes in postural stability became apparent mainly when visual or head-position conditions were altered, assessment under such conditions may provide additional information on postural control in adolescents with idiopathic scoliosis. However, because no healthy control group was included and sensory integration was not directly assessed, these findings should not be interpreted as evidence of AIS-specific sensory-integration deficits. In addition, because the measurement error, test–retest reliability, and minimal detectable change in the assessment system have not been established, the magnitude of the observed differences should be interpreted with caution. These preliminary findings require confirmation in larger, controlled studies including healthy age-matched participants.

## Figures and Tables

**Table 1:** Participant characteristics (mean ± standard deviation).

Variable	Mean ± SD/n
Age (years)	15.3 ± 1.8
Height (cm)	162.5 ± 6.8
Weight (kg)	48.5 ± 6.9
BMI (kg/m^2^)	17.6 ± 1.8
Sex (F/M)	18/1

cm: centimeter; kg: kilogram; BMI: body mass index; SD: standard deviation; F: female; M: male.

**Table 2:** Relative plantar-load distribution under the three testing conditions.

Load Distribution	Eyes Open	p*	Eyes Closed	p*	Head Extension	p*
Right foot load (%)	49.21 ± 2.95 (48 [47.5–51])	*0.30*	48.79 ± 3.61 (48 [46–51.5])	0.18	49.53 ± 4.34 (49 [47–51])	*0.29*
Left foot load (%)	50.63 ± 3.08 (52 [48–52.5])		51.16 ± 3.66 (52 [48–54])		50.74 ± 3.86 (51 [49–53])	
Right MT1 (%)	22.05 ± 8.18 (22 [18–27])	0.085	23.11 ± 8.12 (22 [18–29])	0.056	24.89 ± 9.53 (26 [18.5–29.5])	0.056
Left MT1 (%)	26.00 ± 7.03 (27 [21.5–29.5])		27.42 ± 7.12 (27 [22.5–34])		29.68 ± 7.99 (28 [23–35])	
Right MT5 (%)	33.84 ± 6.53 (34 [28.5–39])	0.001	34.63 ± 7.19 (37 [28.5–40])	0.001	33.84 ± 6.52 (32 [29.5–40])	0.002
Left MT5 (%)	25.05 ± 7.28 (24 [20.5–31.5])		26.11 ± 7.14 (27 [21.5–30.5])		25.63 ± 7.18 (25 [22.5–29])	
Right heel (%)	43.95 ± 8.00 (46 [41.5–48.5])	0.044	42.00 ± 8.42 (42 [39–48])	0.056	41.68 ± 11.63 (45 [31.5–49])	0.085
Left heel (%)	48.84 ± 8.35 (48 [43–54.5])		45.89 ± 7.23 (45 [40–51.5])		45.11 ± 9.97 (49 [36–53])	

Data are presented as mean ± standard deviation with median [interquartile range] in parentheses. MT1: first metatarsal head; MT5: fifth metatarsal head; regional percentages are expressed relative to each foot’s load. p* gives the right-versus-left side comparison; for the regional sites these *p*-values are Holm-adjusted across the regional family (foot-total *p*-values are uncorrected). The fifth-metatarsal asymmetry remained significant after correction in all conditions, and the heel asymmetry only in the eyes-open condition. Values in italics are not statistically significant.

**Table 3:** Stabilometric data in the testing conditions (eyes open, eyes closed, head extension).

Stabilometric Data	Eyes Open	Eyes Closed	Head Extension	*p* ^a^	*p* ^b^	*p* ^c^
CoP path length (mm)	354.7 ± 79.0 (333 [319–354.5])	460.1 ± 162.2 (424 [390.5–482])	418.1 ± 112.5 (444 [372.5–489])	0.011	0.032	0.121
90% confidence-ellipse area (mm^2^)	130.3 ± 200.1 (62 [43.5–120])	196.4 ± 140.8 (164 [73.5–224.5])	186.3 ± 143.4 (131 [71–295])	0.017	0.016	0.441
Maximum CoP speed (mm/s)	75.3 ± 25.2 (70 [63–75.5])	148.4 ± 130.1 (97 [87.5–123])	127.9 ± 96.6 (104 [85–133])	0.001	0.008	0.872

Data are presented as mean ± standard deviation with median [interquartile range] in parentheses; CoP: center of pressure. *p*-values for pairwise comparisons are Holm-adjusted: *p*^a^, eyes open vs. eyes closed; *p*^b^, eyes open vs. head extension; *p*^c^, eyes closed vs. head extension. Friedman omnibus tests: CoP path length χ^2^(2) = 15.47, *p* < 0.001, *W* = 0.41; 90% confidence-ellipse area χ^2^(2) = 13.05, *p* = 0.002, *W* = 0.34; maximum CoP speed χ^2^(2) = 15.89, *p* < 0.001, *W* = 0.42.

## Data Availability

The original contributions presented in this study are included in the article/Supplementary Material. Further inquiries can be directed to the corresponding authors.

## Supplementary Materials

The following supporting information can be downloaded at: https://www.mdpi.com/article/10.3390/jcm15176867/s1, Table S1. Individual raw data for static plantar-load distribution and stabilometric parameters in all 19 participants under the three testing conditions. Regional plantar-load values (MT1, MT5, heel) are expressed as a percentage of each foot’s load, and foot totals as a percentage of total body weight; stabilometric values are absolute.
