# Supplementary material for: Static Plantar-Load Distribution and Stabilometry in Children and Adolescents with Idiopathic Scoliosis Under Different Testing Conditions: A Pilot Study
Source: J Clin Med. 2026 Sep 4;15(17):6867. doi: 10.3390/jcm15176867 (PMC13566573; doi:10.3390/jcm15176867)
Supplement: Supplementary file 1 [file jcm-15-06867-s001.zip › jcm-4435221-supplementary.pdf]

# Supplementary Materials

**Table S1.** Individual raw data for static plantar-load distribution and stabilometric parameters in all 19 participants under the three testing conditions. Regional plantar-load values (MT1, MT5, heel) are expressed as a percentage of each foot's load, and foot totals as a percentage of total body weight; stabilometric values are absolute.

## (a) Eyes open

| Participant | Right foot load (%) | R_MT1 (%) | R_MT5 (%) | R_Heel (%) | Left foot load (%) | L_MT1 (%) | L_MT5 (%) | L_Heel (%) | CoP path length (mm) | 90% ellipse area (mm²) | Max CoP speed (mm/s) |
|---|---|---|---|---|---|---|---|---|---|---|---|
| 1 | 51 | 19 | 36 | 45 | 49 | 28 | 24 | 48 | 346 | 53 | 74 |
| 2 | 46 | 33 | 34 | 33 | 54 | 26 | 31 | 43 | 379 | 45 | 79 |
| 3 | 44 | 25 | 26 | 48 | 56 | 36 | 9 | 55 | 319 | 62 | 63 |
| 4 | 49 | 18 | 29 | 53 | 51 | 18 | 25 | 57 | 271 | 76 | 71 |
| 5 | 54 | 18 | 29 | 53 | 46 | 19 | 23 | 58 | 304 | 56 | 58 |
| 6 | 53 | 17 | 36 | 46 | 47 | 15 | 21 | 64 | 354 | 179 | 70 |
| 7 | 48 | 25 | 32 | 43 | 52 | 28 | 33 | 38 | 271 | 18 | 54 |
| 8 | 47 | 26 | 22 | 51 | 53 | 30 | 16 | 54 | 333 | 67 | 67 |
| 9 | 47 | 22 | 34 | 44 | 53 | 24 | 28 | 48 | 323 | 42 | 63 |
| 10 | 51 | 28 | 43 | 29 | 49 | 29 | 33 | 38 | 330 | 53 | 63 |
| 11 | 50 | 18 | 36 | 46 | 47 | 16 | 20 | 63 | 353 | 178 | 71 |
| 12 | 48 | 28 | 27 | 45 | 52 | 36 | 18 | 46 | 296 | 38 | 57 |
| 13 | 53 | 6 | 44 | 49 | 47 | 24 | 21 | 54 | 331 | 22 | 63 |
| 14 | 51 | 12 | 42 | 46 | 49 | 28 | 27 | 45 | 353 | 100 | 77 |
| 15 | 45 | 33 | 28 | 39 | 55 | 37 | 20 | 43 | 355 | 35 | 83 |
| 16 | 48 | 19 | 41 | 40 | 52 | 15 | 38 | 48 | 319 | 91 | 59 |
| 17 | 54 | 26 | 26 | 48 | 46 | 27 | 23 | 50 | 475 | 899 | 133 |
| 18 | 48 | 9 | 38 | 53 | 52 | 24 | 34 | 41 | 617 | 321 | 152 |
| 19 | 48 | 37 | 40 | 24 | 52 | 34 | 32 | 35 | 411 | 140 | 73 |

## (b) Eyes closed

| Participant | Right foot load (%) | R_MT1 (%) | R_MT5 (%) | R_Heel (%) | Left foot load (%) | L_MT1 (%) | L_MT5 (%) | L_Heel (%) | CoP path length (mm) | 90% ellipse area (mm²) | Max CoP speed (mm/s) |
|---|---|---|---|---|---|---|---|---|---|---|---|
| 1 | 51 | 24 | 40 | 36 | 49 | 29 | 29 | 41 | 483 | 239 | 141 |
| 2 | 46 | 30 | 34 | 36 | 54 | 24 | 30 | 45 | 450 | 73 | 89 |
| 3 | 44 | 28 | 24 | 47 | 56 | 36 | 10 | 54 | 423 | 71 | 90 |
| 4 | 48 | 16 | 31 | 52 | 52 | 20 | 29 | 51 | 136 | 127 | 89 |
| 5 | 56 | 18 | 31 | 51 | 44 | 21 | 25 | 54 | 424 | 164 | 118 |
| 6 | 52 | 19 | 38 | 43 | 48 | 18 | 27 | 56 | 402 | 210 | 103 |
| 7 | 45 | 28 | 30 | 42 | 55 | 28 | 33 | 38 | 379 | 206 | 66 |
| 8 | 46 | 30 | 26 | 44 | 54 | 34 | 16 | 50 | 481 | 161 | 98 |
| 9 | 50 | 22 | 37 | 41 | 50 | 25 | 31 | 45 | 542 | 249 | 389 |
| 10 | 48 | 31 | 42 | 27 | 52 | 34 | 28 | 38 | 378 | 196 | 67 |
| 11 | 51 | 18 | 38 | 42 | 48 | 18 | 27 | 48 | 402 | 210 | 103 |
| 12 | 49 | 21 | 26 | 53 | 51 | 34 | 20 | 45 | 426 | 57 | 83 |
| 13 | 53 | 7 | 46 | 47 | 47 | 25 | 22 | 52 | 410 | 67 | 84 |
| 14 | 52 | 11 | 40 | 49 | 48 | 29 | 26 | 45 | 372 | 74 | 86 |
| 15 | 45 | 33 | 27 | 40 | 55 | 42 | 21 | 37 | 502 | 119 | 128 |
| 16 | 48 | 18 | 41 | 40 | 52 | 17 | 36 | 46 | 913 | 443 | 536 |
| 17 | 53 | 27 | 23 | 50 | 47 | 27 | 16 | 57 | 363 | 519 | 97 |
| 18 | 42 | 18 | 44 | 38 | 58 | 24 | 37 | 39 | 792 | 473 | 363 |
| 19 | 48 | 40 | 40 | 20 | 52 | 36 | 33 | 31 | 463 | 73 | 89 |

## (c) Head extension

| Participant | Right foot load (%) | R_MT1 (%) | R_MT5 (%) | R_Heel (%) | Left foot load (%) | L_MT1 (%) | L_MT5 (%) | L_Heel (%) | CoP path length (mm) | 90% ellipse area (mm²) | Max CoP speed (mm/s) |
|---|---|---|---|---|---|---|---|---|---|---|---|
| 1 | 51 | 23 | 28 | 49 | 49 | 30 | 20 | 50 | 457 | 198 | 98 |
| 2 | 46 | 26 | 29 | 45 | 54 | 22 | 25 | 53 | 373 | 59 | 69 |
| 3 | 47 | 30 | 23 | 47 | 53 | 39 | 8 | 53 | 380 | 56 | 88 |
| 4 | 47 | 15 | 31 | 54 | 53 | 20 | 28 | 52 | 54 | 65 | 67 |
| 5 | 53 | 20 | 32 | 49 | 47 | 19 | 24 | 57 | 466 | 314 | 501 |
| 6 | 57 | 17 | 31 | 53 | 43 | 21 | 23 | 56 | 524 | 574 | 114 |
| 7 | 49 | 29 | 30 | 41 | 51 | 32 | 32 | 37 | 303 | 78 | 68 |
| 8 | 49 | 27 | 30 | 42 | 51 | 32 | 15 | 52 | 444 | 97 | 133 |
| 9 | 49 | 27 | 44 | 28 | 51 | 28 | 30 | 41 | 479 | 129 | 104 |

| | | | | | | | | | | | |
|---|---|---|---|---|---|---|---|---|---|---|---|
| **10** | 49 | 36 | 42 | 22 | 51 | 37 | 28 | 35 | 415 | 156 | 89 |
| **11** | 59 | 14 | 29 | 64 | 46 | 27 | 24 | 58 | 372 | 389 | 82 |
| **12** | 47 | 26 | 27 | 47 | 53 | 34 | 23 | 42 | 351 | 75 | 73 |
| **13** | 56 | 5 | 42 | 54 | 44 | 23 | 22 | 55 | 356 | 55 | 133 |
| **14** | 49 | 16 | 46 | 38 | 51 | 41 | 27 | 33 | 499 | 181 | 189 |
| **15** | 42 | 43 | 32 | 25 | 58 | 48 | 22 | 30 | 503 | 67 | 138 |
| **16** | 51 | 27 | 41 | 32 | 49 | 28 | 40 | 32 | 567 | 313 | 174 |
| **17** | 49 | 20 | 35 | 45 | 51 | 23 | 28 | 49 | 425 | 277 | 90 |
| **18** | 43 | 30 | 39 | 31 | 57 | 24 | 33 | 43 | 527 | 326 | 110 |
| **19** | 48 | 42 | 32 | 26 | 52 | 36 | 35 | 29 | 449 | 131 | 110 |

Abbreviations: MT1, first metatarsal head; MT5, fifth metatarsal head; CoP, center of pressure. Participants 1–16 and 17–19 correspond to the two acquisition batches in the source dataset (a blank separator row in the original spreadsheet has been removed); both batches were acquired with the same PoDATA 2.0 platform, software version, calibration procedure, assessor, standardized protocol, and examination room. Data audit: all values were verified against the original PoDATA 2.0 device reports. Cell E2 of the eyes-closed sheet (left-foot load of participant 1, initially recorded as 196) was identified through a bilateral-sum consistency check and corrected to the value in the original device report (49); no other values required correction. Because foot and regional loads are exported as separately rounded integer percentages, bilateral foot-load sums may deviate from 100% by a few percentage points; these deviations reflect rounding rather than data errors. Participants 6 and 11 are two different individuals; their identical stabilometric values under the eyes-closed condition were re-checked against the original device reports and confirmed to be correct.
